# A method for a categorized and probabilistic analysis of the surface electromyogram in dynamic contractions

**DOI:** 10.3389/fphys.2015.00030

**Published:** 2015-02-11

**Authors:** Sylvie C. F. A. Von Werder, Tim Kleiber, Catherine Disselhorst-Klug

**Affiliations:** ^1^Department of Rehabilitation & Prevention Engineering, Institute of Applied Medical Engineering, RWTH Aachen UniversityAachen, Germany; ^2^German Research School for Simulation Sciences, Joint Graduate School of RWTH Aachen University and Forschungszentrum JülichJülich, Germany

**Keywords:** sEMG, dynamic, probabilistic, categorization, contraction type, joint angle

## Abstract

The human motor system permits a wide variety of complex movements. Thereby, the inter-individual variability as well as the biomechanical aspects of the performed movement itself contribute to the challenge of the interpretation of sEMG signals in dynamic contractions. A procedure for the systematic analysis of sEMG recordings during dynamic contraction was introduced, which includes categorization of the data in combination with the analysis of frequency distributions of the sEMG with a probabilistic approach. Using the example of elbow flexion and extension the procedure was evaluated with 10 healthy subjects. The recorded sEMG signals of brachioradialis were categorized into a combination of constant and variable movement factors, which originate from the performed movement. Subsequently, for each combination of movement factors cumulative frequency distributions were computed for each subject separately. Finally, the probability of the difference of muscular activation in varying movement conditions was assessed. The probabilistic approach was compared to a deterministic analysis of the same data. Both approaches observed a significant change of muscular activation of brachioradialis during concentric and eccentric contractions exclusively for flexion and extension angles exceeding 30°. However, with the probabilistic approach additional information on the likelihood that the tested effect occurs can be provided. Especially for movements under uncontrollable boundary conditions, this information to assess the confidence of the detected results is of high relevance. Thus, the procedure provides new insights into the quantification and interpretation of muscular activity.

## Introduction

Muscles move us and many scientists move the research on the interpretation of muscular activation. Thus, muscular activation is investigated and described in a wealth of literature to increase the knowledge of physiological movement patterns or pathological movement disorders. But, the human motor system permits a wide variety of complex movements. To meet the challenge of controlling all possible movements, the human motor system exhibits redundant muscular activation strategies ranging from strategies on how to activate one muscle to strategies on activating a consortium of contributing muscles. The study of muscular activation with surface Electromyography (sEMG) provides insights into the contribution of single muscles to the overall performed movement. Like the human motor system itself, the interpretation of muscular activation is complex. There are controversial results from examination of sEMG as well as reviews on the limitation of sEMG. The interpretation of sEMG recordings requires caution (Rau et al., 2004) especially in dynamic tasks (Farina, 2006). The sEMG is dependent on several factors, which impact on the relation between the recorded sEMG and the underlying physiological process. Farina et al. divided the factors that influence the sEMG in two groups, “non-physiological” and “physiological” factors (Farina et al., 2004). Amongst others, they noted the shape and thickness of the investigated muscle and surrounding tissue, the electrode position and inter-electrode distance, crosstalk of nearby muscles, as well as fiber membrane and motor unit properties, which can affect the recording and consequently the interpretation of the sEMG. Furthermore, individual differences due to the muscular condition or habitual motion patterns might affect the sEMG and contribute to the challenge of interpreting dynamic sEMG recordings.

But, especially dynamic tasks are of high importance for considerations on pathological movement disorders. At this point, daily living activities which involve free movements of the limbs are restricted. On the one hand, analyzing free movements further challenges the interpretation of the dynamically recorded sEMG. On the other hand, possible sEMG findings under highly controlled movement conditions in a physiological reference cannot be easily transferred to the pathological situation of patients. Sometimes the patients are not able to move their limbs under the same highly controlled conditions, e.g., patients with spasticity after stroke. Thus, the balance between investigating freely performed movements on the one hand and the possibility to interpret the dynamic recorded sEMG on the other hand is intended. Considering the example of spasticity, the muscular activation can be exclusively recorded in the range of the patients' movement capability, leading to uncontrollable boundary conditions of the investigated movement. Consequently, the performed movement is more complex just as the interpretation of the sEMG. However, the interplay of the mechanisms, which contribute to the human motion, is to the same extent of physiological relevance as the controllable single physiological mechanisms themselves. Especially if pathologies are involved, the interplay of several factors is of high relevance.

If less restricted and uncontrollable movement conditions are inevitable, specific analyzing techniques are needed to assess the confidence of the detected results. In this context is it possible to provide additional information on the likelihood of the occurrence or absence of an investigated physiological or pathological effect?

In this manuscript two aspects on the analysis of dynamic sEMG recordings are considered: the impact of the performed movement and the procedure of the performed analysis of dynamic sEMG recordings.

Regarding the impact of the performed movement itself, several factors have been described which influence the muscular activation and originate from the way in which the performed movement was conducted. Considering the elbow flexion and extension movement, the effect of muscular contraction type on the activation of contributing elbow muscles was evaluated. In that context, a decreased activation during eccentric contraction depending on the joint angle and pre-activation mode was observed (Komi et al., 2000; Linnamo et al., 2006). Additionally, differences of angle dependent variations of the synergistic activation of biceps brachii and brachioradialis during eccentric contractions were found, which were not observed during concentric contractions (Nakazawa et al., 1993). Besides the performed movement itself, there is an inter-individual variability of sEMG profiles (Guidetti et al., 1996; Hug, 2011).

Regarding the procedure of the performed analysis, the way of the interpretation of the processed data is crucial. Once the data is processed it can be assessed with a deterministic approach or with a probabilistic approach. For a deterministic approach the data is successively examined for each time point, to compute mean values for the performed movement cycles as it was performed for the muscular activation patterns of the gait analysis (Perry, 1992). For a deterministic approach, the processed sEMG is utilized as input to compute for a fix output, e.g., the mean ± standard deviation. In contrast, for a probabilistic approach, probabilities are assigned to the occurrence of specific events. Instead of comparing sEMG means ± standard deviation, a probabilistic approach assesses frequency distributions of the sEMG recordings to compute probabilities that the analyzed sEMG recording takes a specific value. In that way the examined signal, with an uncertain nature of the sEMG recording itself is assessed with a fuzzy approach instead of crisp deterministic values. A probabilistic method was described to estimate the levels of muscular activation for the functional electrical stimulation (Anderson and Fuglevand, 2008) and the control of neuroprosthetics (Johnson and Fuglevand, 2009).

Taking the example of flexion and extensions of the elbow, this report describes a procedure to analyze sEMG recordings of dynamic contractions with a systematic categorization of the recorded data combined with a probabilistic approach. In that way the described procedure aims to account for the impact of the performed movement on the one hand and moreover aims to add an uncertain component to the interpretation of the recorded sEMG on the other hand to provide information on the confidence of the obtained results. The probabilistic approach was compared to a deterministic analysis of the same data.

## Materials and methods

The procedure to systematically analyze sEMG recordings of dynamic contractions was introduced using the example of elbow flexion and extension with respect to the muscular activation of brachioradialis.

### Concept of systematic analysis

Before analyzing the muscular activation in dynamic contraction possible influencing factors of the intended movement must be extracted. In the case of elbow flexion and extension, several factors which influence the muscular activation and origin from the type of the performed movement are known. These factors include the impact of the joint position, meaning the supinated, neutral or pronated forearm position, or the impact of concentric or eccentric contraction types. Furthermore, the external force as well as the velocity of the performed movement might impact on the muscular activation. Each movement factor must be assigned to remain either constant or variable during the intended movement of the study. If one factor remains constant during the experiment, the interpretation of the data is limited to this constant factor and consequently all constant movement factors must be indicated.

As a result the data can be recorded in defined constant or variable conditions of influencing factors, which originate from the performed movement. Subsequently, the raw data is processed and categorized into a combination of influencing movement factors. For each categorized data a relative frequency distribution is computed to assign probabilities of the muscular activation in defined dynamic movement conditions, which are further assessed in a probabilistic manner.

### Participants

The experiments were conducted with 10 healthy subjects (age 23 ± 3 years; height 179 ± 9 cm; body mass 75 ± 8 kg). No subject had any known symptoms of neuromuscular disorders, history of orthopedic surgery or any affection of the upper extremities. Subjects avoided strenuous exercises in the day prior to the measurement. All subjects were right handed. All experiments were performed in accordance to the declaration of Helsinki and all subjects gave written informed consent prior to the study.

### Experimental setup

The experimental setup included the measurement of bipolar sEMG and elbow joint angles. sEMG-electrodes, with an inter-electrode distance of 2 cm (Hermens et al., 2000), were placed on the brachioradialis. For the placement of the electrodes the lower arm was moved to neutral forearm position. The subject was asked to perform an elbow flexion against resistance at the wrist. The electrodes were placed at the level of one third from the fossa cubit along the palpable muscle belly. sEMG signals were sampled at a frequency of 2000 Hz. Kinematics of elbow flexion and extension movement were recorded with a VICON MX (10 cameras) motion capture system with a sampling rate of 200 Hz and an electrogoniometer (Biometrics Ltd) with a sampling frequency of 2000 Hz. The marker setup included five joint markers and four triplets of segment markers. The joint markers were placed at the radial and ulnar styloid process of the wrist, the epicondyle lateral and medial at the elbow and the acromion. The segment markers were placed at the hand, forearm, upper arm and thorax.

Regarding the influencing movement factors, the joint position, the external force and the performed velocity were kept constant. The subjects were measured in standing position with anatomical hand posture, representing a neutral forearm position, with the shoulder in neutral rest position, meaning being neither flexed nor abducted. The subjects had to flex and extend their elbow with an external weight of 3.5 kg and a constant speed of 25°/s. The elbow flexion and extension angle as well as the contraction type were assigned as variable influencing movement factors. At this point, it must be marked that although the weight that the subject had to carry was constant at 3.5 kg, the external moment due to this weight changed with the flexion and extension angles. Therefore, the angle impact is dependent on the amount of force produced to move the constant external weight.

In order to prevent fatigue, measuring intervals were limited to one repetition of flexion and extension followed by 60 s of relaxation time. A minimum of 20 repetitions of full flexion and extension movements of the elbow joint were performed to gather enough data for the subsequent probabilistic analysis (Anderson and Fuglevand, 2008; Johnson and Fuglevand, 2009). The flexion and extension course recorded with the electrogoniometer was visualized to provide a feedback on the constant velocity performance of the subjects. For normalization a set of five maximum voluntary contractions (MVC) in 90° of elbow flexion and neutral forearm position were performed prior to the elbow flexion and extension measurements. Between each MVC trial a break of a minimum of 180 s was made to avoid the possibility of fatigue.

### Signal processing

Signal processing of the kinematic data included computation of flexion and extension as well as pronation and supination angles. The marker trajectories were filtered with a spline filter of third order (Schmidt et al., 1999). With the help of a biomechanical model of the human arm the pronation and supination angles of the elbow were computed (Rau et al., 2000; Williams et al., 2006). All data sets with a change of pronation and supination angles exceeding ±5° were excluded from further analysis to ensure that the subjects remained their constant joint position and avoided any pro- or supinating movement of the elbow. Likewise, any data sets with movement of the adjacent joints, namely the shoulder and wrist, exceeding ±10° were excluded.

The sEMG recordings were band pass-filtered (10–500 Hz), rectified and smoothed (moving average filter, window length 100 ms) in order to compute the envelope of the sEMG signal. Normalization of the envelope was performed with respect to the MVC. For that purpose the mean value of the enveloped sEMG of the recorded three MVC trials was computed. Out of five MVC trials with constant torques the mean of the three MVC sEMG envelopes with the smallest standard deviation is chosen as reference.

### Categorization of the processed data

**Figure 1:**
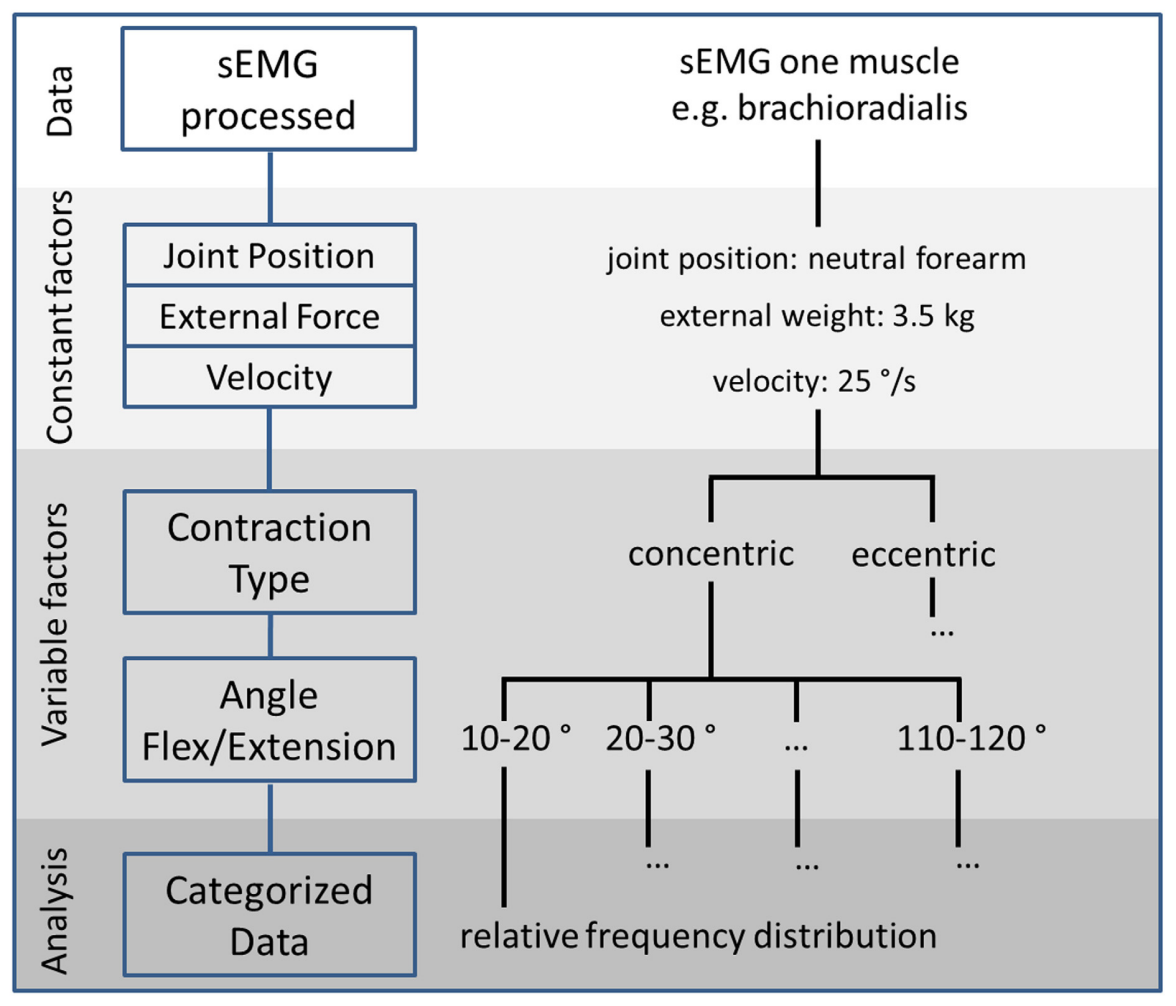
**A decision tree is used to categorize the processed sEMG data into a combination of constant and variable movement factors to finally analyze relative frequency distributions of the sEMG with respect to all possible movement factors**.

A decision tree was used to distinguish the present combination of variable movement factors for each sampling point of the recorded sEMG data. When the decision algorithm computes the combination of variable movement parameters, the amplitude of the enveloped and normalized sEMG value is stored with an assignment to this combination (Figure 1).

In the example of flexion and extension of the elbow, combinations of the contraction type and flexion and extension angle were assigned to the normalized sEMG value. First, the decision algorithm derived the concentric or eccentric contraction type for the investigated sampling point. Thereby, the contraction types were distinguished in two categories, concentric and eccentric contractions, which were derived from the slope of the flexion and extension course. For the flexing muscles, positive slopes representing flexion of the joint were assigned to concentric contractions, whereas negative slopes representing extension of the joint were assigned to eccentric contractions. Subsequently, the decision algorithm assigned the flexion and extension angle at this sampling point to an angle interval. Here, the decision tree merged the angles into 12 intervals with 10° steps ranging from 0 to 120°, where 0° was full extension.

The categorized data allocates the muscular activation to each combination of variable movement factors and was further assessed with a probabilistic approach.

### Frequency distribution for probabilistic analysis

**Figure 2:**
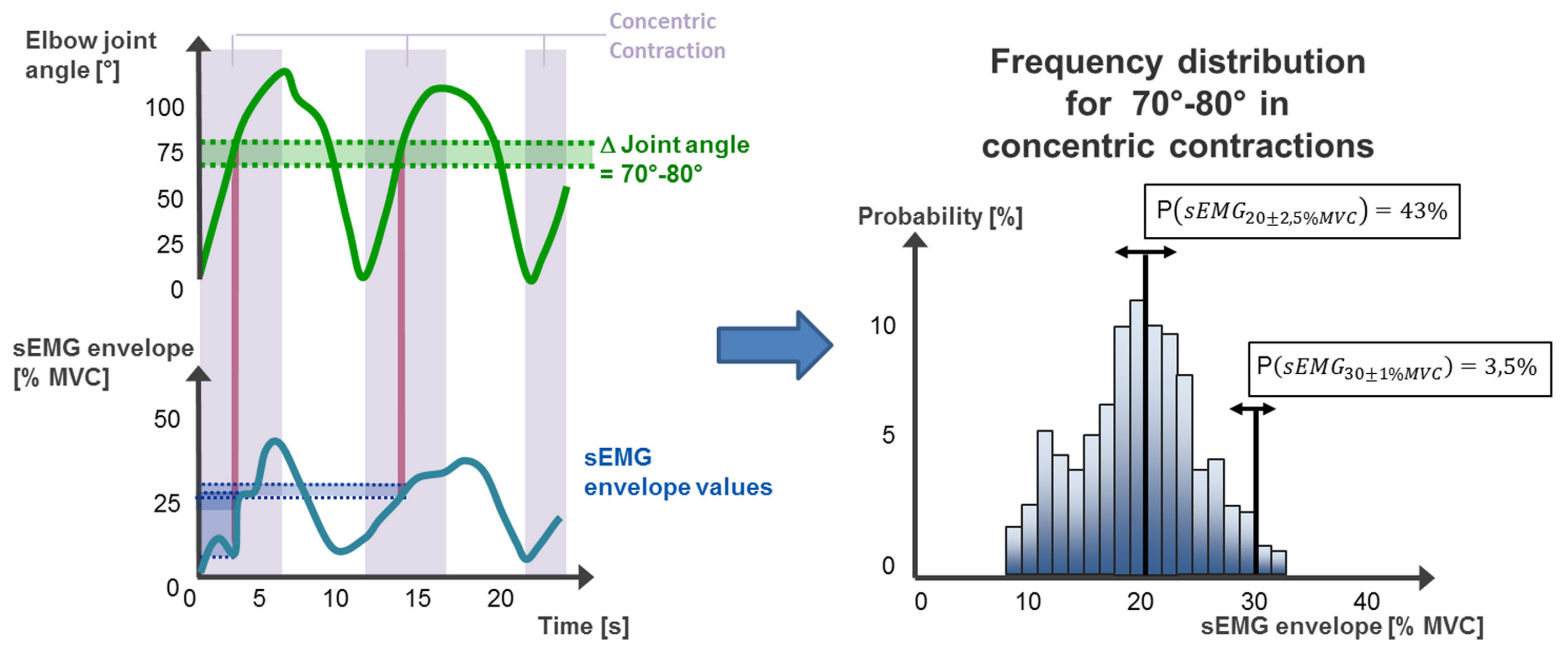
**For each sampling point the value of the sEMG envelope is assigned to a combination of variable movement parameters**. A relative frequency distribution of the corresponding sEMG is computed for each category of contraction type and flexion and extension angle. Additionally, the probability of sEMG envelope to be within a defined interval can be computed.

The impact of the variable movement factors of flexion and extension angle and contraction type for the combination of constant movement factors was further assessed with the help of a probabilistic approach. All amplitudes of the enveloped and normalized sEMG in one combination of angle category and contraction type were evaluated to derive a relative frequency distribution for each subject separately. From that relative frequency distribution a cumulative frequency can be computed to derive the probability for each possible sEMG value to be within a defined interval (Figure 2).

### Evaluation of categorized data analysis

To demonstrate the relevance of assigning possible movement parameters to the sEMG signals, the recorded sEMG signals of brachioradialis of all trials and all subjects were analyzed in three different analyzing scenarios in a deterministic way. For that purpose, for the same data the normalized sEMG envelopes were categorized with three different decision trees to compute mean values ± standard deviations of all trials and all subjects. The results were further analyzed for statistical relevance.

The first analyzing scenario A included a decision tree with the contraction type as variable movement parameter only, without any respect to the different elbow flexion and extension angles. Exclusively the contraction type was used to categorize the processed sEMG signal into two contraction types.

The second analyzing scenario B included a decision tree with the variable movement parameter flexion and extension angles without any respect to the contraction type. Exclusively the flexion and extension angles were used to categorize the processed sEMG into 12 intervals ranging from 0 to 120°. For the statistical analysis the angle intervals were merged into four intervals with four times 30° steps.

The third analyzing scenario C included the described decision tree with the combination of two variable movement parameters namely the combination of 12 angle and two contraction type intervals. For statistical analysis angle intervals were merged into four intervals with four times 30° steps.

### Probabilistic analysis of the categorized data

The assignment of probabilities for each possible categorized sEMG value to be within a defined interval enables the test of the impact of movement factors on the muscular activation. Thus, the categorized frequency distributions of the normalized sEMG envelope were used for further analysis. To demonstrate the procedure, the following hypothesis was exemplarily tested.

The muscular activation is affected by a combination of contraction type and flexion and extension angle.

**Figure 3:**
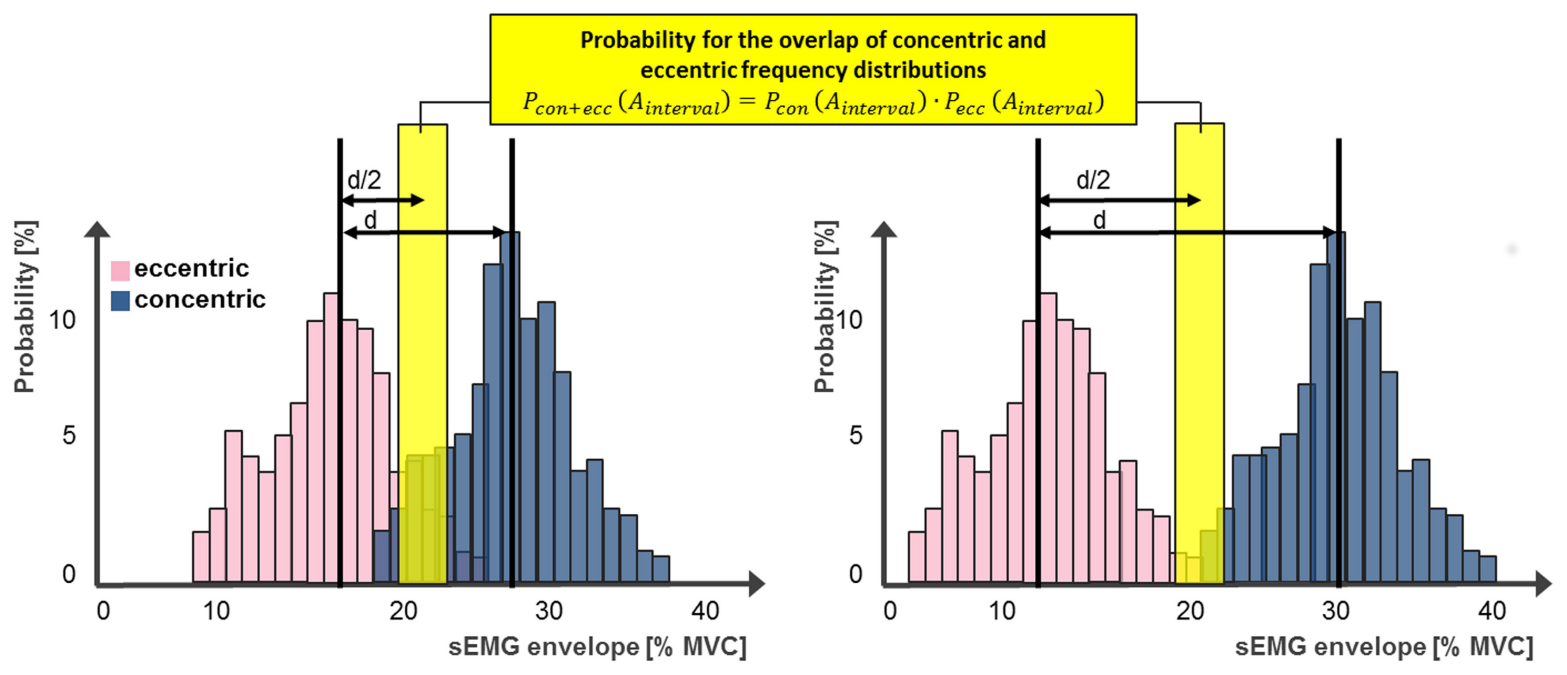
**Two examples for the procedure on the evaluation of two frequency distributions**. The probability of overlap of concentric and eccentric frequency distributions of the same angle interval can be computed through the multiplication of the relative frequencies of each distribution to contain sEMG values in a defined analyzing interval. This interval is located at d/2 in the middle between the most probable normalized sEMG envelopes of each distribution. If two distributions distinguish from each other, the probability of overlap would be zero.

For the test of the raised hypothesis, the frequency distributions of normalized sEMG envelopes in concentric and eccentric contraction were assessed in the same four flexion and extension angle intervals as performed in analyzing scenario C. For that purpose a probability for each angle interval was computed for each subject as described in the following (Figure 3).

For each subject the distance d between the most probable normalized sEMG envelope of the frequency distributions during concentric and eccentric contractions were computed for each angle interval separately. In the middle between the most probable normalized sEMG envelope of the frequency distributions of concentric and eccentric contractions d/2 an analyzing interval A_interval_ was defined.

(1) $${\text{A}}_{\text{interval}}\;=\;\frac{\text{d}}{2}\;\pm \;2.5\%\text{ MVC}$$

Subsequently, the probability of the normalized sEMG envelope to occur within the defined analyzing interval was computed for both contraction types separately, as concentric P_con_ (A_interval_) and eccentric P_ecc_ (A_interval_) cumulative frequencies.

The probability that the value of normalized sEMG envelope of concentric and eccentric contractions occurs in both frequency distributions at the defined interval P_con + ecc_ (A_interval_) was computed through the multiplication of both cumulative frequencies:

(2) $$P_{con\;+\;ecc}\left(A_{interval}\right)\;=\;P_{con}\left(A_{interval}\right)\cdot P_{ecc}\left(A_{interval}\right)$$

The more the frequency distributions of concentric and eccentric contraction apart from each other, the smaller the probability for an overlap of both distributions at the defined interval gets.

To compare the probabilistic approach to a deterministic analysis the probabilities for the overlap of concentric and eccentric contractions in all four angle intervals of each subject were computed. Subsequently, the probabilities of each subject in one angle interval were averaged and finally compared to the deterministic outcome of the same categorized data of test scenario C.

### Statistical analysis

All statistical analyses were performed with SPSS (SPSS Inc., Chicago, IL, USA). The level of statistical significance for all analyses was set to α = 0.05.

For the categorized data of scenario A a paired-samples *t*-test was used to determine whether there was a statistically significant difference between the categorized normalized sEMG envelopes (dependent variable) during concentric contractions compared to eccentric contractions. Test for normality was performed with Shapiro-Wilk's test.

For scenario B an One-Way repeated measures ANOVA was conducted to determine whether there were statistically significant differences of the normalized sEMG envelope (dependent variable) over the course of four different angle conditions (within subject factor). A *Post-hoc* analysis with a Bonferroni adjustment was used to compare the change of the normalized sEMG envelope with increasing angles. Normal distribution and sphericity of the categorized data was assessed by Shapiro-Wilk's test and Mauchly's test of sphericity, respectively.

For scenario C a Two-Way repeated measures ANOVA was run to determine the effect of four different angle conditions (within subject factor 1) in two different contraction types (within subject factor 2) on the normalized sEMG envelope (dependent variable). Subsequently, simple main effects on the contraction type were run to assess the difference of normalized sEMG in concentric and eccentric contractions under all angle conditions separately. Again, normal distribution and sphericity of the categorized data was assessed by Shapiro-Wilk's test and Mauchly's test of sphericity, respectively.

## Results

The described method for the analysis of dynamic sEMG signals assigns the muscular activation to movement parameters originating from the way of the performed movement. Thereby, the results are limited to the constant movement parameters, the constant velocity of 25°/s and the constant external weight of 3.5 kg. Data are mean ± standard deviation, unless otherwise stated.

First, the effect of categorization of the data was tested for the described analyzing scenarios. For the first analyzing scenario A the contraction type was found to take a significant impact on the muscular activation of brachioradialis. A statistically significant difference between normalized sEMG envelope of concentric contractions compared to eccentric contractions were found [*t*_(9)_ = 5.256, *p* = 0.001] with a higher normalized sEMG during concentric contractions (19.14 ± 7.30% MVC) compared to eccentric contractions (13.76 ± 5.62% MVC). The difference of the concentric and eccentric normalized sEMG envelopes of the categorized data was normally distributed, as assessed by Shapiro-Wilk's test (*p* = 0.703).

For the second analyzing scenario B the flexion and extension angle showed a significant impact on the normalized sEMG envelope. The categorized normalized sEMG envelopes of scenario B were normally distributed at each angle condition, as assessed by Shapiro-Wilk's test (*p* > 0.05). Mauchly's test of sphericity indicated that the assumption of sphericity in test scenario B had been violated, χ^2^ = 20.489, *p* = 0.001. A Greenhouse-Geisser correction (ε = 0.401) (Greenhouse and Geisser, 1959) was applied to correct the One-Way repeated measures ANOVA. The different angle conditions elicited statistically significant changes of the mean normalized sEMG envelope [*F*_(1.204, 9.633)_ = 39.793, *p* < 0.0005] with the normalized sEMG envelope increasing from 4.67 ± 2.28% MVC for flexion angles of 0–30° to 23.90 ± 10.55% MVC for flexion angles of 90–120°. *Post-hoc* analysis with a Bonferroni adjustment revealed that there was a statistically significantly increase from 0–30° to 30–60° (*p* < 0.0005), from 30–60° to 60–90° (*p* = 0.005), but not for 60–90° to 90–120° (*p* = 0.485).

Analysis of the studentized residuals of the categorized normalized sEMG envelopes of scenario C showed that there was normal distribution, as assessed by the Shapiro-Wilk test (*p* > 0.05). Additionally, there was sphericity for the interaction term, as assessed by Mauchly's test of sphericity (*p* > 0.05). A statistically significant interaction between contraction type and angle condition was found [*F*_(3, 27)_ = 24.732, *p* < 0.0005]. The simple main effects on the contraction type revealed that the normalized sEMG envelope was statistically significantly different for the concentric compared to the eccentric contractions at the angle condition of angles between 30 and 60° [*F*_(1, 9)_ = 21.743, *p* = 0.002], 60–90° [*F*_(1, 9)_ = 27.803, *p* = 0.001], and 90–120° [*F*_(1, 9)_ = 36.070, *p* < 0.0005]. However, the normalized sEMG envelope was not statistically significantly different for the concentric compared to the eccentric contractions at the angle condition of angles between 0 and 30° [*F*_(1, 9)_ = 0.022, *p* = 0.539] (**Figure 5**).

**Figure 4:**
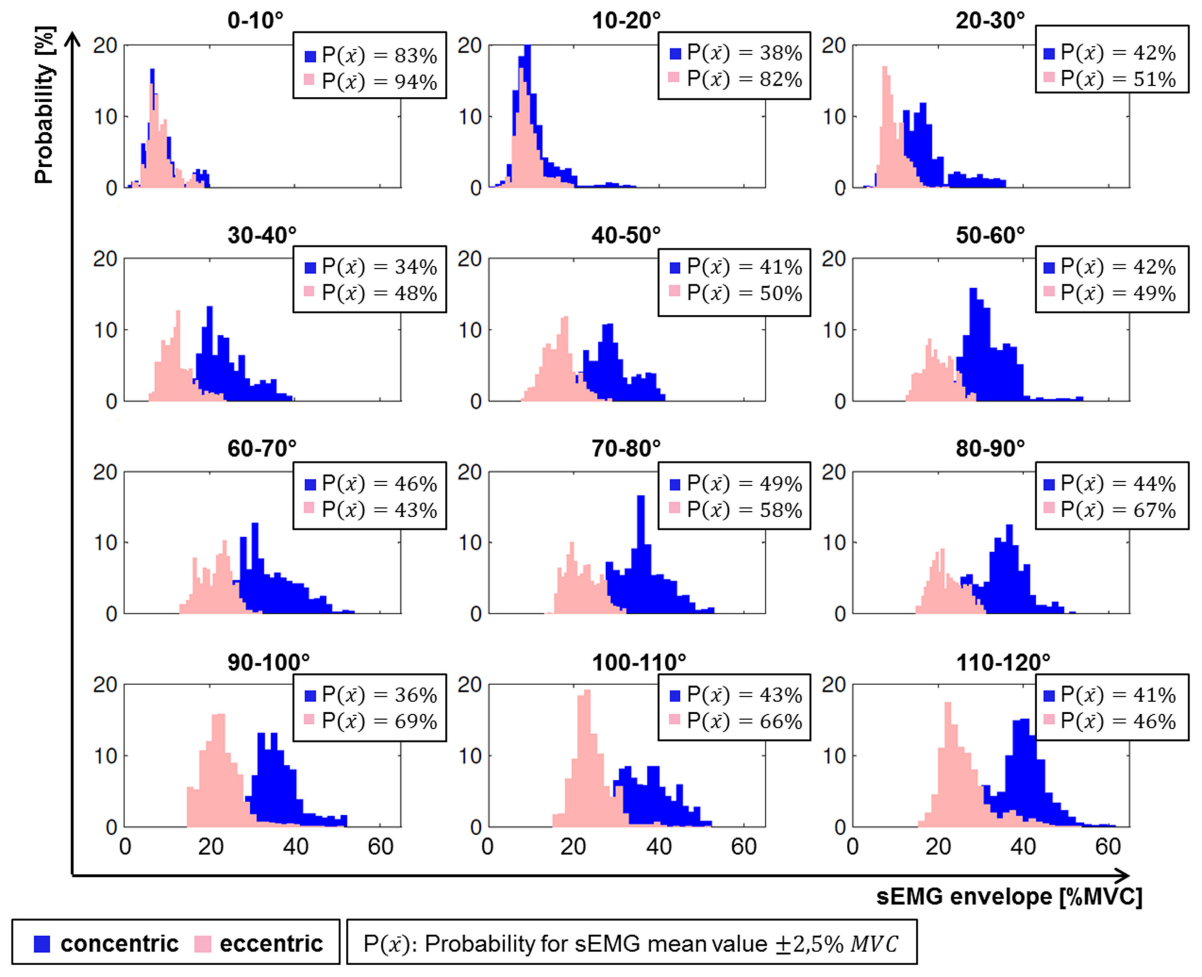
**Activation profiles of one subject of concentric (blue) and eccentric (pink) contractions of brachioradialis during different elbow flexion and extension angles with the information on the probability of the mean value of sEMG envelope to remain within ±2.5% MVC interval**.

The probabilistic analysis of scenario C supported that the combination of both movement parameters impacts on the muscular activation. Exemplarily Figure 4 illustrates the frequency distribution of the categorized sEMG of brachioradialis of one subject. The combination of contraction type and flexion and extension angle can be assigned to a change of the processed sEMG signal. For small flexion and extension angle no difference of the most probable normalized sEMG envelope for eccentric and concentric contractions was found. With increasing angles the most probable normalized sEMG envelope was found to be higher in concentric contraction compared to eccentric contractions.

The results of the probabilistic analysis refer to the parameter of the probability for overlap of concentric and eccentric frequency distributions at the defined interval in all four angle intervals separately. For angles between 0 and 30° the median of the probabilities for the overlap of concentric and eccentric frequency distributions for all recorded subjects was found to be 69.33%, with a range of 33.04–90.73%. For angles exceeding 30° the probability for the overlap of concentric and eccentric frequency distributions at the defined interval decreased (**Figure 6**). The decreased probabilities represent that the frequency distributions of concentric and eccentric contractions are more likely to differ from each other. For 30–60°, 60–90°, and 90–120° the median of the probability for overlap decreased to 5.77% with a range of 0.01–24.98%, 4.88% with a range of 0.02–24.24%, 5.37% with a range of 0.05–28.82%, respectively (Table 1).

**Table 1:** **Overview on the deterministic and probabilistic results of the categorized data of analyzing scenario C**.

**Angle interval [°]**	**Deterministic approach Two Way repeated ANOVA**		**Probabilistic approach probability overlap [%]**	
	***F*_(1,9)_**	***p*-value**	**Median**	**Range**
0–30	0.022	0.539	69.33	33.04–90.73
30–60	21.743	0.002	5.77	0.01–24.98
60–90	27.803	0.001	4.88	0.02–24.24
90–120	36.070	<0.0005	5.37	0.05–28.82

The deterministic as well as the probabilistic approach observed a significant change of muscular activation of brachioradialis during concentric and eccentric contractions exclusively for flexion and extension angles exceeding 30° (Table 1).

## Discussion

The analysis of sEMG in dynamic contractions contains multiple factors which impact on the analysis of the data and consequently might affect the possible interpretations. Sometimes the analysis of muscular activation during dynamic and complex movements is of high relevance, in particular if pathological movements are to be evaluated. Thereby, less restricted and uncontrollable movement conditions are inevitable and lead to a more complex interpretation of the sEMG.

The introduced procedure for the systematic analysis of sEMG recordings during dynamic contraction includes a categorization of the data combined with the analysis of frequency distributions in a probabilistic manner.

Independently on a deterministic or probabilistic analysis of dynamic contractions, the first step must be assigned to a systematic categorization of the data. Through the categorization of the data with the help of a decision tree, the possible findings on muscular activation patterns were assigned to a combination of movement parameters. The three analyzing scenarios revealed that in the example of flexion and extension of the elbow, the muscular activation of brachioradialis is affected by the variable movement factors contraction type and flexion and extension angle. From the analyzing scenario A one can conclude that concentric contractions need a higher normalized sEMG envelope amplitude than eccentric contractions. Additionally, from the analyzing scenario B one can conclude that the amplitude of normalized sEMG increases with increasing flexion and extension angle. However, analyzing scenario C highlights that not the assignment of one movement factor is sufficient, but the combination of both movement factors is important whenever changes in the muscular activation of brachioradialis are detected. Although analyzing scenario A showed for all subjects an impact of contraction type on the muscular activation of brachioradialis, for small angle intervals no difference of the normalized sEMG amplitude for eccentric and concentric contraction were observed. Therefore, the categorization of the data and the assignment to constant movement parameters is important when dynamic sEMG recordings are evaluated. However, to avoid extremely complex measuring setups, not all movement parameters must be defined as variable movement factors. It is reasonable to assign possible findings to a combination of variable and constant movement factors. But it is essential to highlight the constant movement factors, since the possible findings are limited to them.

**Figure 5:**
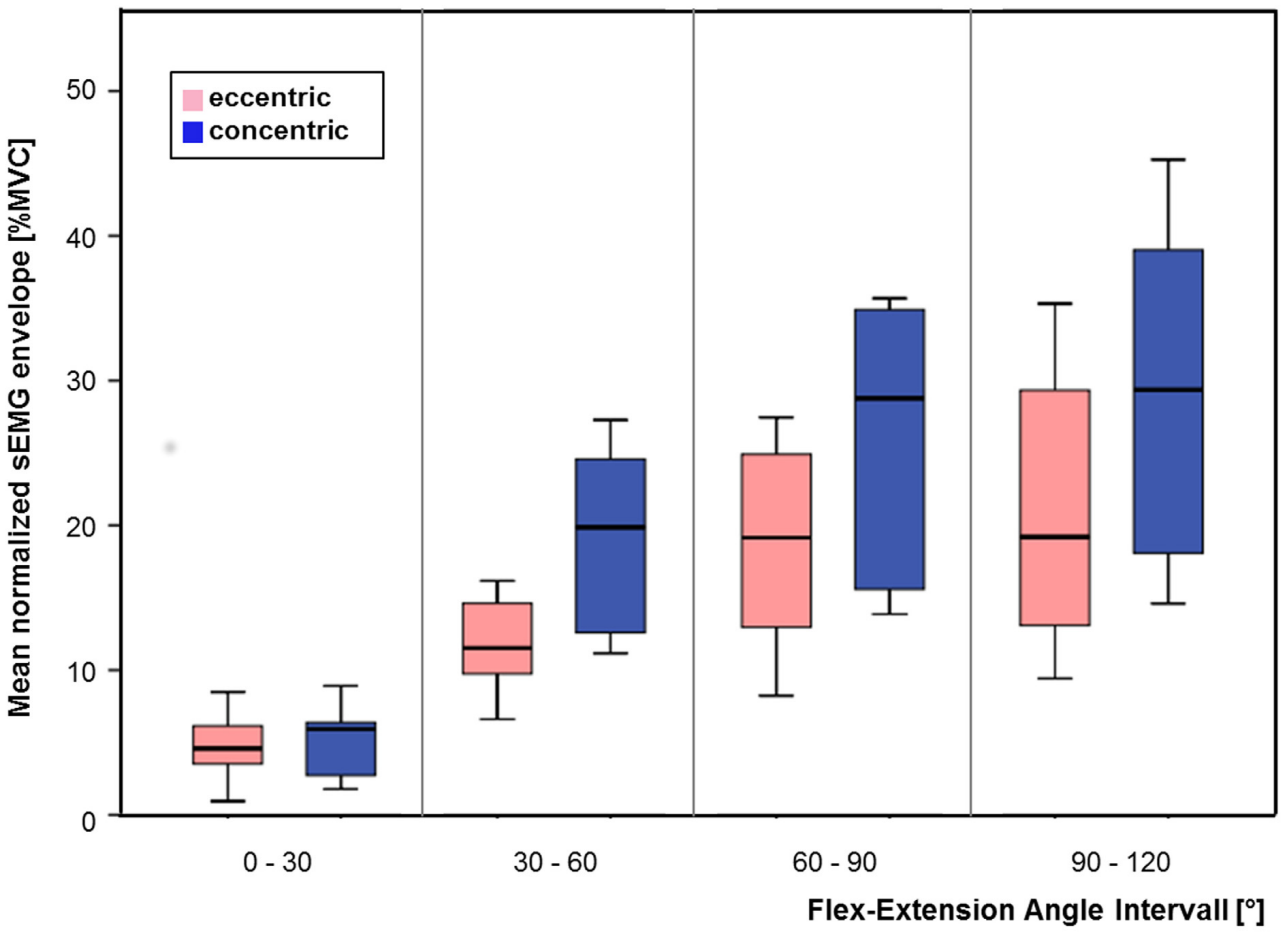
**Mean values of sEMG envelope in four different elbow flexion angle intervals with concentric (blue) and eccentric (pink) contractions of brachioradialis**.

**Figure 6:**
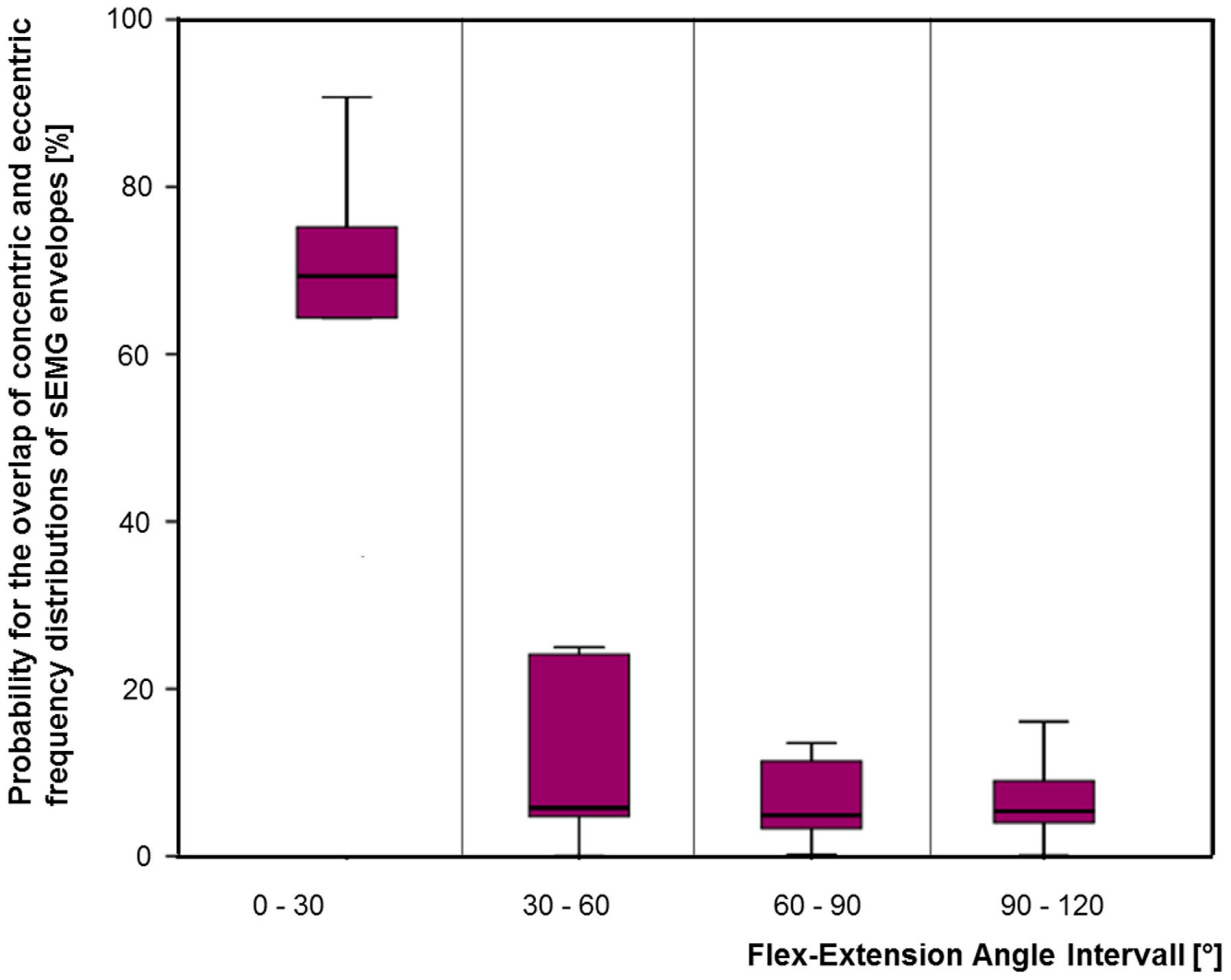
**The probability that the frequency distributions of concentric and eccentric categorized data overlaps within an interval of 2.5% MVC was computed for four flexion and extension angle intervals**.

For the comparison of the deterministic approach (Figure 5) to the probabilistic approach (Figure 6), summarized in Table 1, it can be concluded that both approaches reveal the same effect in that the concentric and eccentric activation of brachioradialis differ from each other for angles exceeding 30°. However, whereas the deterministic analysis computes for a statistical significant effect, the probabilistic approach provides additional information on the probability that the same effect does or does not occur. The result from the repeated two way measure indicate a statistical difference between concentric and eccentric contraction for angles exceeding 30°, (*p* = 0.002, *p* = 0.001 and *p* < 0.0005 for angles between 30–60°, 60–90°, and 90–120°, respectively). In contrast, the probabilistic parameter for the overlap of both frequency distributions provides additional information on the likelihood that this difference is not present. The decreased probabilities for angles exceeding 30° represent that the frequency distributions of concentric and eccentric contractions are more likely to differ from each other. But at the same time, the value of the probable overlap in a defined interval accounts for the probability of the negative effect, namely that the concentric and eccentric sEMG do not differ from each other. For angles between 30 and 60° the median and range of the probability that both frequency distributions in a defined interval do not differ from each other was computed to 5.77% with a range of 0.01–24.98%. Thereby, in four of the 10 subjects the probability exceeded 20%, meaning that for these subjects the tested hypothesis, with respect to the parameter of overlap, holds true with a probability of less than 80%. With the probabilistic parameter additional information are provided to add a confidence to the obtained results. The ranges of the computed probabilities for the overlap of both frequency distributions reveal that there are inter-individual differences for the activation of brachioradialis in concentric and eccentric contractions along all angle areas.

For the deterministic analysis the outcome of the statistical test is to reject or hold the raised hypothesis. In contrast, if the analysis is connected to the test of a specific hypothesis with a probabilistic approach, additional information on the likelihood that the hypothesis holds true or fails are available which adds a confidence to the results. More probabilistic methods could be added to the evaluation of two frequency distributions. Besides the similarity or difference of two frequency distributions one could also assess the probability that one sEMG distribution possess higher sEMG amplitudes than the other.

The main potential of the probabilistic approach lies in the fact that each possible value of the normalized sEMG can be assigned to a probability that this value will occur in a specific interval. In combination with parameters, which describe the similarity or difference of two frequency distributions, the information on the likelihood that the analyzed effect occurs or not occurs can be provided. It can be assumed, that inter-individual differences increase with more uncontrollable movement conditions in free movements. A measure on the confidence of the detected results could account for this increasing effect of inter-individual differences.

Analyzing sEMG signals during dynamic contractions is challenging. However, the introduced procedure of analyzing categorized data with a probabilistic approach allows to address the challenge of the influence of different movement conditions on the muscular activation and provides additional information on the confidence of the results by adding probabilities to the tested hypothesis.

### Conflict of interest statement

The authors declare that the research was conducted in the absence of any commercial or financial relationships that could be construed as a potential conflict of interest.
